# Sexual health-related care needs among young adult cancer patients and survivors: a systematic literature review

**DOI:** 10.1007/s11764-021-01084-w

**Published:** 2021-10-20

**Authors:** Vicky Lehmann, Ellen T. M. Laan, Brenda L. den Oudsten

**Affiliations:** 1 Department of Medical Psychology, Amsterdam University Medical Center, Cancer Center Amsterdam, Meibergdreef 9, 1105 AZ Amsterdam, Netherlands; 2 Department of Sexology and Psychosomatic Gynecology, Amsterdam University Medical Center, Meibergdreef 9, 1105 AZ Amsterdam, Netherlands; 3 Department of Medical and Clinical Psychology, Center of Research On Psychological and Somatic Disorders, Tilburg University, Tilburg, Netherlands

**Keywords:** Sexual health, Cancer (survivorship) care, Young adult oncology, Needs, Information, Communication

## Abstract

**Purpose:**

Young adult cancer patients and survivors have age-specific care needs, but care needs related to sexual health are poorly understood. A systematic literature review was conducted to examine sexual health-related care needs among patients/survivors diagnosed with cancer during young adulthood (age: 18–39 years). The prevalence and types needs were assessed along with associated patient factors.

**Methods:**

Four major databases were screened to identify relevant studies, which were also assessed for risk of bias; all following PRISMA guidelines.

**Results:**

Identified studies (*N* = 35) often assessed sexual health-related care needs by whether participants experienced a generic need for support from providers. The prevalence of such needs ranged between 8 and 61.7% and was higher in female survivors and those with more health impairments. The type of diagnosis could also play a role in these varying prevalence rates, but was not systematically tested in included studies. Types of sexual health-related care needs were clustered into practical/emotional support needs (e.g., coping with physical side effects), information needs (e.g., more details), and communication needs (e.g., providers should initiate conversations, validate concerns, be empathetic/open). Needs should be addressed in-person and/or online.

**Conclusions:**

The extent of needs related to sexual health varies among young adult patients and survivors, but types of needs center around improving provision of support and information by providers.

**Implications for Cancer Survivors:**

Sexual health should routinely be addressed alongside other potential effects of cancer treatment to allow for constructive conversations between patients and providers. Referrals to (online) resources or specialists should be tailored to individual preferences.

**Supplementary Information:**

The online version contains supplementary material available at 10.1007/s11764-021-01084-w.

## Introduction

Cancer treatment and its side effects can cause various sexual problems, including decreased sexual interest and activity, arousal problems, diminished feelings of attractiveness, pain, vaginal dryness, or erectile dysfunction, all contributing to impaired sexual functioning [1–7]. Yet, attention to sexual health in clinical practice during and following cancer treatment is limited. Providers and patients do not prioritize sexual health in the light of a cancer diagnosis, and discussing sexual health can be uncomfortable for both [8–17]. Providers and patients can also have an implicit bias toward neglecting sexual health if patients are single and/or older [11, 18]. Although the elderly may be overlooked in clinical care, ample research on sexual function has focused on survivors of breast and prostate cancer [19, 20], who are typically diagnosed well above the age of 50. In contrast, those being diagnosed during young adulthood (age 18–39 years) sometimes feel like a “lost tribe” [21], because education materials or interventions are often not tailored to them. There is consensus that young adults with cancer have age-specific needs [22–25], which should be addressed appropriately in clinical care [26], but practical implementations remain vague, and care depends on the dedication of individual providers.

Next to an overrepresentation of older cancer survivors in sex-related research, previous studies also often focused on patients with types of cancer that have a direct impact on sex, such as breast, prostate, testicular, or other reproductive organ-related cancers. However, young adults can be diagnosed with any type of cancer, which can directly or indirectly affect their sex lives, given that emerging and young adulthood is a life stage marked by various developmental tasks (e.g., engaging in more serious relationships and possibly starting a family [27, 28]). In clinical practice and interview studies, young adults with cancer expressed feelings of suddenly being faced with “old-people’s problems” (e.g., menopausal symptoms or erectile problems), which they feel they cannot discuss with same-aged peers [29]. Patients and survivors can also have other age-specific difficulties that may negatively affect their sex lives, such as dealing with a young beauty ideal in the light of an impaired body image, fertility problems, caring for young children in the home, more aggressive treatment regimens that diminish energy levels, social disruptions in experimenting with their sexuality, disclosure of cancer, and (online) dating new partners during and following cancer treatment [2, 30–36].

Overall, clinical cancer care and research has begun to understand the unique challenges and their complex effects on young adult patients’ and survivors’ sex lives. Accordingly, healthcare providers are encouraged to address sexual health with young adult cancer patients and survivors [37–40], but such well-intended recommendations often lack a comprehensive empirical basis. At the same time, intervention programs are now being developed, which focus on young adults [41–43] and specifically address sexual health [44, 45]. However, such programs tend to emphasize fertility over sexual health, and not every patient or survivor needs a structured intervention program.

Thus, from recognizing sexual problems toward potentially offering interventions, we still miss a crucial step in-between: understanding what young adult patients/survivors think they need from providers to address their sexual health. Thereby, it is also important to try and identify who might be in need the most. For example, different treatment modalities can affect sexual functioning differently [1, 46], but whether related care needs are also diverse and specific for certain types of treatment or diagnoses remains unknown. This systematic review aims to fill these gaps by summarizing the existing literature and identifying (i) the prevalence of sexual health-related care needs (i.e., any sex-related supportive/ healthcare need) and whether prevalence rates are linked to patient or clinical characteristics (e.g., sex, type of diagnosis or treatment). Moreover, (ii) the types of needs that should be addressed by providers will be examined. The findings of this review will help facilitate an evidence-based approach to addressing sexual health-related care needs among young adults with cancer in clinical practice.

## Methods

### Literature search and eligibility

This review followed the PRISMA guidelines for conducting and reporting reviews [47]. Four major databases (Medline, PsycInfo/PsycArticles, EMBASE, CINAHL) were searched using terms that included a combination of cancer-, sex-, young-, and needs-related search terms in their title or abstracts (see Online Resource 1 for a full overview). To further ensure a thorough approach, the reference lists of all included papers were screened as well.

The search identified a total of 1519 records, published before July 2020. After duplicate extraction, 844 unique records were retained, and their titles and abstracts were screened. A random selection of 25% (*n* = 211/844) were screened by two authors (VL, BdO), who reached an excellent inter-rater agreement of 93%. Citations that were identified by both or either author were retained for full-text review. All remaining citations were screened by one author (VL). Subsequent full-text reviews to assess the final inclusion of papers were done by one author (VL), but any uncertainties were discussed to reach consensus.

Studies were included if they (a) reported any kind of need related to sexual health or intimacy that may require addressing by healthcare providers (e.g., general support, information). This also implies that studies were excluded if they focused on informal/peer support or if they only assessed the presence of impaired sexual functioning/sexual problems or fertility-related concerns. Eligible studies also (b) focused on needs based on self, provider, or partner report of patients/survivors diagnosed with cancer during young adulthood (i.e., age at diagnosis: 18–39 years). If studies included participants with broader age ranges at diagnosis, they were retained if (b.1) the mean age was within the young adult age range or if (b.2) they reported subgroup results for young adults (i.e., excluding studies where outcomes could not be delineated for young adults). Please note that this criterion implies that studies which recruited adolescents and young adults (AYAs) with a mean age < 18 and/or that reported no subgroup results for young adults were excluded. While certainly interesting, we argued that there are crucial differences between adolescents and young adults when it comes to addressing their sexual health (e.g., maturity/puberty, treatment at a pediatric vs. adult facility), and most importantly, young adults are legally allowed to make medical decisions and can request sexual health-related counseling without parent consent, all further underlining the focus of this review. Eligible studies also had to (c) present original data (i.e., excluding reviews, commentaries, or study protocols), which also required reporting results of (d) more than *n* = 1 participant (i.e., excluding case reports or single quotes in qualitative studies). Note that although reviews and study protocols were excluded from this review, they were marked during screening and checked for potentially eligible papers. Finally, eligible studies had to be (e) written in English.

### Study outcomes and quality assessment

Study findings of care needs related to sexual health will be presented. The following information was extracted and entered into pre-defined tables and SPSS-sheets: year and place of study, sample size, sex, participant age at study, age at diagnosis, type of diagnosis (if mixed, the most common type was added), used instruments/methods, type of data (i.e., qualitative, quantitative), reported findings of sexual health-related care needs, and the focus of the study. Response rates were (re-)calculated for each study, if possible. A full overview of such information for each included study is presented in Online Resource 2.

Study findings will be reported based on our two aims. First, prevalence rates of sexual health-related care needs will be reported in percentages. Trends based on participant characteristics will be summarized (e.g., sex, type of diagnosis, treatment). Second, study findings will be used to cluster types of reported needs. This has been done utilizing an approach similar to thematic content analysis, which descriptively presents qualitative data [48, 49]. Traditionally, one would use interview data and continuously cluster reoccurring uttered topics and themes. Similarly, we clustered reoccurring themes at study level. For example, reports of needing, wanting, or missing information were clustered together. Moreover and if reported, the kind of needed information was also registered (and eventually added to this cluster), while we continuously checked for no overlap with other clusters (e.g., needing information vs. manner of information provision; see “Results” section). All data were analyzed and clustered by one author (VL) and critically discussed among all.

We also intended to assess the quality of all included studies, but most studies were not designed to specifically focus on sexual health and/or to recruit young adults (see below). Often, only parts of reported data and/or subgroups were extracted for this review. Therefore, we deemed structured quality assessment tools (e.g., [50–52]) as unfeasible and sometimes unfair to assess the included studies. For example, assessing whether a study used an appropriate design to answer their research question does not allude to whether the extracted findings related to sexual health were of good quality. Another example is that assessing the representativeness of included participants would be unfair and sometimes impossible if only subgroup results are reported here. Instead, we present several indicators of potential risks of bias, which are also used in quality ratings (e.g., low response rate, sample size, methods), along with other points of consideration for each study (Online Resource 3).

## Results

### Study inclusion

**Fig. 1:**
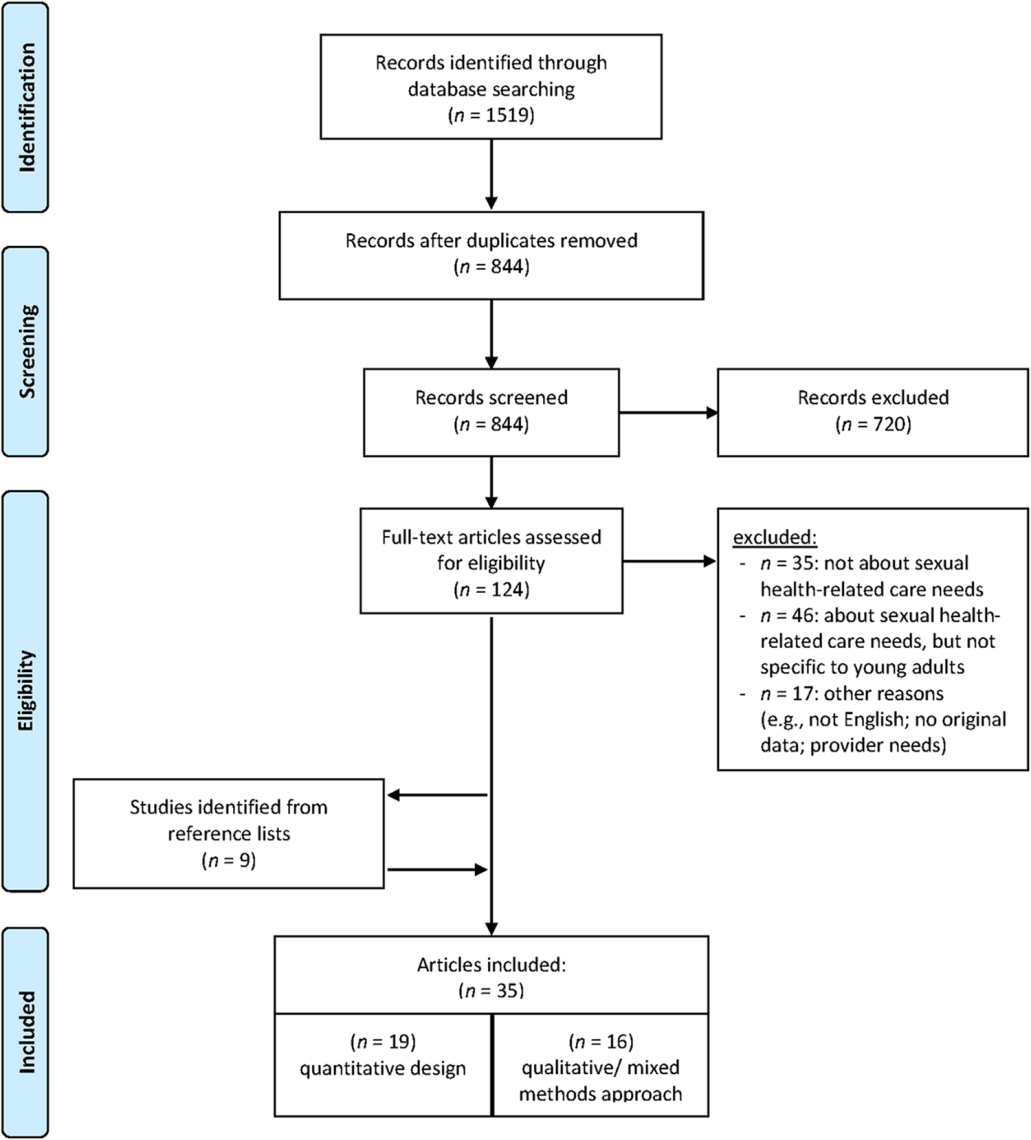
PRISMA flowchart of study inclusion and exclusion

The titles and abstracts of all *N* = 844 unique citations were screened for eligibility, resulting in *n* = 124 references for full-text review. Of these full texts, *n* = 35 were excluded for being unrelated to sexual health-related care needs, and *n* = 46 were on topic, but did not recruit or report results specific to young adults. Another *n* = 17 articles were excluded for other reasons (see PRISMA flow chart in Fig. 1). This resulted in *n* = 26 eligible studies, and their reference lists were screened for other potentially relevant articles. Additionally, the content and reference lists of 19 reviews and 21 study protocols/intervention papers were screened. These had been marked during the initial title/abstract screening as being somewhat related to sexual health, but did not meet inclusion criteria. From all reference lists, a total of *n* = 246 citations were reviewed, which yielded *n* = 9 additional manuscripts (Fig. 1).

A total of *N* = 35 studies were included in this review, which had recruited *n* = 8–879 young adult participants (total *N* = 5938). Studies used a quantitative design (*n* = 19) or qualitative/ mixed methods approach (*n* = 16), and most had been published within the last decade of 2010–2020 (80%, *n* = 28/35). The majority of studies included patients and survivors with mixed types of cancer diagnoses (60%, *n* = 21/35) or focused on those diagnosed with breast (*n* = 8), gynecological (*n* = 4), or testicular cancers (*n* = 2). Most studies either included women only (*n* = 12/35; 34%) or recruited more than 60% female participants (*n* = 9/35; 26%), whereas male participants were exclusively recruited in *n* = 3 studies (9%).

Few studies (*n* = 6/35, 17%) focused specifically on sexual health and related care needs, and all but one study had at least one potential risk of bias indicator (Online Resource 3). Assessments of sexual health-related care needs were based on self-developed survey items, self-developed interview questions/ focus group guides, a 3-item subscale of the Supportive Care Needs Survey (SCNS [53, 54]), or a single item of the Cancer Survivors’ Unmet Needs Measure (CaSUN [55]). For example, self-developed survey items included face-valid questions like the occurrence and need for sex-related discussion [34, 56], need for support [57], or need for counseling/guidance related to sexuality and intimacy ([24]; see Online Resource 2 for exact item wording). The three SCNS items assess unmet needs of healthcare providers’ sensitivity to “changes in sexual feelings,” “changes in sexual relationships,” and “giving information about sexual relationships” [53, 54]. The CaSUN-item assesses whether participants experience unmet needs regarding their “sex life” ([55]). Thus, identified studies assessed rather generic perceptions of sexual health-related care needs that should be attended to by providers (see below; Online Resource 2).

### Prevalence of needs

A total of *n* = 10 studies assessed the presence of generic support needs related to sexual health among patients and survivors of cancer [24, 34, 56–63], which ranged between 8 and 61.7% (Table 1). Studies included samples of 50–879 participants and typically comprised participants with mixed types of cancer diagnoses (*n* = 7/10). The three largest studies, which included 514–879 survivors, reported the highest rates (49.5–61.7% [24, 34, 61]). Lowest rates were reported by a study that directly compared long-term survivors of testicular cancer and lymphoma [57], where 8% of *n* = 50 lymphoma and 21.5% of *n* = 264 testicular cancer survivors reported sexual health-related support needs (Table 1).

**Table 1:** *S*tudies that reported prevalence rates of sexual health-related support needs (*n* = 10) among young adult cancer patients/survivors (in descending order of reported rates; see Online Resource 3 for the potential risk of bias in each study)

1st author Year Location	N	Sex	Diagnosis (most common)	Age at/time since diagnosis	Age at study	Measure^a^	Reported prevalence rates of sexual health-related care needs
Graugaard 2018 [34] Denmark	822	51% Female	Mixed (melanoma)	*M*_age_ = 24, 15–29 *M*_time_ = 3.9 years, 1–7	28, 17–36	Self-developed	- Sex-related discussions during follow-up: 61.7% unmet needs - Sex-related discussions during hospitalization: 49.5% unmet needs
Sender 2019 [61] Germany	514	75% Female	Mixed (breast)	*M*_age_ = 30 (6.1), 18–39 *M*_time_ = 12.1 months	nr	SCNS-SF34 [54]	- 49.6% at baseline - 46.9% 1 year later
Zebrack 2009 [24] USA	879	72% Female	Mixed (Hodgkin)	*M*_age_ = 26 (5.8), 15–35	30 (8.1), 18–39	Self-developed	- 40.2% needed counseling/ guidance (which remained unmet for 73.7% [= 30% of total sample])
Geue 2015 [59] Germany	99	66% Female	Mixed (hematological)	Age: 15–39 *M*_time_ = 30 months	33 (5.6), 18–45	1 SCNS-item: changes in sexual feelings	- 38.3%
Bender 2012 [58] Canada	204	100% Male	Testicular	*M*_time_ = 2.2 (1.1) years	36 (10.5)	Adapted CaSUN-item	- 37% (not in top 10 met or unmet needs)
Hall 2012 [60] Australia	58	71% Female	Mixed (melanoma)	*M*_age_ = 35, 18–40 *M*_time_ = 7 months	18–40	SCNS-SF34 [54]	- 29.3%
Smith 2013 [62] Australia	244	100% Male	Testicular	*M*_age_ = 35, 16–69 *M*_time_ = 2.3 (< 5) years post treatment	38 (10.3), E21–68	CaSUN-item [55]	- 23% (2^nd^ most common need)
Jonker Pool 2004 [57] Netherlands	264, 50	100% Male	Testicular (lymphoma)	*M*_age_ = 29/34 (17–70) *M*_time_ = 5.9 years	36/42	Self-developed	- 21.5% testicular cancer vs. 8% lymphoma survivors
Zebrack 2013 [63] USA	111	53% Female	Mixed (breast)	41% aged 20–29 59% aged 30–39 years *M*_time_ = 66 days	31 (6.0)	Needs questionnaire [24]^b^	- Counseling/guidance related to sexuality/intimacy: - Age 20–29: 14.6% unmet need - Age 30–39; 21.5% unmet need
Kedde 2013 [56] Netherlands	332	100% Female	Breast	< 6 years; 43% completed treatment	39, 22–49	Self-developed	- 15.2% unmet needs (i.e., 50.6% reported sexual dysfunction, of whom 50% ever perceived a need for sex-related care, but 60% did not receive it)

^a^See Online Resource 2 for more details on self-developed items; ^b^ later used as “needs questionnaire” [24]; *nr* not reported, *SCNS-SF* Supportive Care Needs Survey (short form), *CaSUN* Cancer Survivors’ Unmet Needs Measure

Moreover, an interview study including 35 breast cancer survivors [64] described unmet needs “related to sexual issues” among only 3% of survivors, but these needs were not described or defined (see also risk of bias in Online Resource 3). At item level, three studies reported sex-related needs as not being included in the top 10 or top 20 most common unmet needs [58, 65, 66], including one study among testicular cancer survivors [58]. In contrast, another rather similar study among testicular cancer survivors reported it as the second most common unmet need item [62], which is also the only included study with no identified risk of bias indicator (Online Resource 3). On the subscale level, one study among mixed types of cancer survivors reported that “sexuality care needs” ranked third out of five domains [67].

#### Trends in prevalence rates

##### Diagnosis, treatment, and time since diagnosis

Three of the above studies in breast cancer survivors reported particularly low rates of sexuality-related needs, that is, 15.2% [56], 3% [64], or not a top 10 unmet need [66]. In contrast, three studies, focusing on testicular cancer survivors, reported moderate rates of 21.5–37% of survivors having sexual health-related care needs [57, 58, 62], which was also considerably higher than the 8% rate among lymphoma survivors [57]. Other trends across studies could not be identified, and except the study comparing testicular cancer and lymphoma survivors [57], other studies did not test differences between patients/survivors with different types of diagnoses.

Effects of treatment type on support needs were tested in one study [57] that reported significantly higher needs among long-term testicular cancer survivors who had been treated with “polychemotherapy” (21.5%; polychemotherapy + surgery: 28.0%) versus those who had received radiation (17.0%) or were put on a surveillance protocol (10.5%). Yet, subsamples were small (*n* = 6–34), and these different treatment regimens were linked to disease stages (i.e., more severe treatment regimens for more severe stages of the disease).

No study systematically tested potential effects of varying time since diagnosis or effects of ongoing versus completed treatment status on sexual health-related care needs. Clues come from a large-scale German-based study that showed no change in needs over a 1-year period as reported by 514 short-term survivors (49% vs. 47% after 1 year [61]). Yet, a study among 822 Danish survivors [34] suggests that care needs may increase following treatment completion: Needs for sex-related discussions were unmet during follow-up care among 61.7% of survivors, whereas such discussions during hospitalization were (retrospectively) perceived as unmet by 49.5% of survivors. Another study asked participants to rate the importance of information and support *during* treatment [68], where counseling related to sexuality or intimacy was rated with a score of 6.3 on a 10-point scale, whereas highest ratings were observed for information on healthy life style or fertility [68].

##### Sex and age

In four studies, which included 99–879 survivors with mixed types of diagnoses [24, 34, 59, 69], female survivors reported significantly more sexuality care needs than males. Sex differences were moderate to large, as indicated by *d* = 0.45 [59] or care needs being 2 times [34] or 1.5 times [24] more common in women. One of these studies (*n* = 577) further identified that among female survivors, those with reproductive organ cancers reported higher sexuality care needs than women with other types of diagnoses (*d* = 0.26), but such difference was not found among men [69]. In another study, ratings of the importance of counseling about sexuality/intimacy did not differ between male and female survivors [68].

Two qualitative studies in female survivors indicated that young adult women experience more unmet sexual care needs than those aged 50 and older [70, 71]. In a similar vein, a quantitative study identified significantly more unmet sexuality care needs among young adult patients than a sex- and cancer type-matched sample of adults age 64 and older (29.3 vs. 10.7% [60]). Another study among recently diagnosed patients specified that 21.5% of those in their 30 s, but only 14.6% of those in their 20 s, experienced unmet care needs regarding sexuality [63].

##### Health

Two large studies in short- to long-term survivors indicated that those with a decreased health status [24] or lower illness adjustment [61] reported more sexuality care needs. Moreover, fatigue was identified as being related to increased care needs among survivors [59].

### Types of sexual health-related care needs

#### Practical/ emotional support needs

In most qualitative/mixed-methods studies (*n* = 12/16), participants voiced a generic need for sex-related services and support, given that such services were absent, scarce, inadequate, and/or not age-appropriate [64, 72–82]. It was specified that patients/survivors need tailored [77] and age-specific support [78], as they missed “skilled and timely interventions” that focus on sex and body image [81]. Young adult survivors also need support to cope with physical side effects of cancer that influence their sex lives (e.g., menopause, vaginal dryness [73]). They further specified needing support to communicate about sex with partners, such as learning how to assert oneself sexually and how to discuss sex openly.

Thereby, relationship status can determine needs, given that single women uttered concern and need for support regarding dating new partners [75, 80], whereas others highlighted the need to include existing partners [73, 74] or to provide services for couples together [74]. Overall, young survivors experienced unmet needs due to providers not prioritizing sexual well-being or due to survivors themselves neglecting their sexuality ([73], see Table 2).

**Table 2:** Overview of types of sexual health-related care needs identified in included studies

Type and specifications	Focus/ topics
**Practical/emotional support needs**	
- Generic need for services and support: - Tailored - Age-specific - Skilled and timely - Support to cope - Refer to specialists (if indicated)	- Sexual well-being - Sex and body image - Physical side effects and implication for sex - Sexual communication with partners - (Online) dating new partners
**Information needs**	
	- On coping with sexual difficulties - To address libido, vaginal dryness - Sexuality, fertility, reproductive issues - Treatment-related effects on body - How to relate to oneself sexually - Dating, new relationships - Body image
**Communication needs**	
Providers should:	
- Initiate discussions - Ensure privacy/time alone with patient/survivor - Validate concerns - Show empathy - Be sensitive, open, not uncomfortable - Be prepared to talk about sexual health - Overcome taboos - Be mindful of cultural aspects, stigma, embarrassment	
Type of preferred personnel	
- Nurse practitioners - Sexologists - (Oncologists)	
Provide information	
- Online - Face-to-face (accommodate personal preferences) - Topical workshops/webinars - Educational materials (e.g., pamphlets)	

#### Information needs

Nine qualitative studies identified a need for additional or more detailed sex-related information among young adult patients and survivors of cancer [70, 71, 73, 75, 76, 80, 82–84], of which only four specified the content of such needed information. It included information on coping with sexual difficulties [71], increasing sexual arousal and reducing related problems (e.g., vaginal dryness [80]), or information from healthcare providers about reaching normative sexual milestones [73]. Moreover, female survivors in a focus group study highlighted that many of their questions remained unanswered [75], as they needed information about treatment-related effects on the body (e.g., fertility, early menopause), as well as information on “how to relate to themselves sexually following treatment, how to contemplate dating, or how to talk about their cancer in new relationships” ([75], Table 2).

Four quantitative studies [57, 76, 83, 85] echoed findings on generic information needs by assessing their presence. These were indicated by 67% of testicular [57] and 52% of breast cancer survivors [76]. In contrast, less than one-third (31%) of survivors with mixed types of cancer [85] and 27% of lymphoma survivors [57] reported such information needs.

The importance of meeting information needs in survivorship about “sexuality, fertility, and reproductive issues” was ranked third highest by young adult survivors [83]. This domain included information about pregnancy safety, options for having a family, menopausal symptoms, and/or genetic risks for offspring [83].

#### Communication needs

Information needs appeared to be closely linked to providers’ communication behaviors. Participants in several studies emphasized that they needed providers to initiate discussions about sexual health [71–73, 76, 84], as survivors described a “silence” surrounding sex [75]. One of these studies included only 8 participants [72], but provided detailed insights: Providers mentioned which physical late effects patients/survivors could expect, but neglected to discuss the implications for sexual health. Survivors also uttered a need for providers to validate any sex-related concerns, to show empathy, and to be sensitive when discussing sexual health [72]. Other studies further highlighted the need to overcome taboos in discussing sexual health [76], the need for privacy/time alone with providers to discuss sensitive matters [82, 86], and the need for providers to be open [79, 86] and prepared to talk about sexual health [82]. It was particularly counterproductive if providers were uncomfortable discussing sex-related matters, such as masturbation [77].

Manners to communicate sex-related information could be online and/or face-to-face [74, 86, 87], whereby providers could also help clarify information that patients/survivors found online [84]. Survivors emphasized that there is “no one size fits all solution” and support provision should be tailored to personal preferences [74]. Nurse practitioners or sexologists had been identified by survivors as most suitable to have sex-related discussions, whereas oncology care providers identified physicians or nurse practitioners as responsible [86]. Patients/survivors also proposed that providers could offer topical workshops [75] or webinars [74] to address lacking information or offer educational materials (e.g., pamphlets [79]; Table 2).

When communicating about sex-related issues, other personal factors may become relevant. For example, cultural background and race/ethnicity can be important, as African American survivors specifically mentioned taboos in their community when discussing sex with providers [76], but cultural and other background factors were not systematically tested in the included studies. Notably, one qualitative study (*n* = 20) recruited equal numbers of White and other racial/ethnic survivors and did not identify any trends [80]. Another study among Chinese cervical cancer survivors [88] reported that the vast majority of young adults (75%) had an interest in sex-related counseling, but 63% would not seek help due to embarrassment or prejudice/stigma [88]. Finally, some survivors find it difficult to initiate or have any sex-related discussions with providers of the opposite sex [71, 86].

## Discussion

This review presents a long overdue summary of care needs related to sexual health among patients and survivors diagnosed with cancer during young adulthood. The 35 identified studies varied in their approaches to assessing sexual health-related care needs and often focused on generic support needs, which were reported by 8–67% of patients/survivors and varied by sex, health status, potentially by cancer type, and time since diagnosis. Types of sexual health-related needs were clustered into practical and emotional support needs, information needs, and communication needs that ought to be tailored to individual patients and survivors (Table 2).

The assessment of sexual health-related care needs in identified studies differed, partly due to many self-developed survey questions, and the assessment remained rather generic as sexual health was not the focus of most studies. A need for support was frequently indicated, but it varied, and the meaning and interpretation of such needs may differ between studies and between individual participants. The largest studies identified the highest prevalence rates (> 40% [24, 34, 61]), offering some confidence in the representativeness of their findings, but studies were not without potential risk of bias (see Online Resource 3). Importantly, these rates were reported by survivors of mixed types of diagnoses, which implies that sexual health can be relevant for various young adult patient/survivors, and not only for those diagnosed with cancers that directly affect sexual organs.

Trends in reported prevalence rates included higher needs among female patients/survivors [24, 34, 59, 69], and women with reproductive organ-related cancers reported somewhat higher needs, whereas such difference was not found among male survivors [69]. Underlying reasons for sex differences and whether differences also exist in expressing needs remain to be examined. Moreover, eight studies exclusively recruited female patients/survivors of breast cancer, but most (*n* = 6/8) were qualitative, and only three studies reported rates of sexual health-related care needs. These were particularly low (< 15.2%), but potential biases might play a role (Online Resource 3), and firm conclusions about the prevalence of sexual health-related care needs in patients/survivors of breast cancer cannot be drawn. In contrast, the three studies in exclusively male participants all recruited patients/survivors of testicular cancer and indicated moderate support needs ranging between 21.5 and 37% (vs. 8% of male lymphoma survivors). Interestingly, one of these studies identified sex-related needs among 37% of testicular cancer survivors, while this item was neither included in the top 10 met nor top 10 unmet need items [58], whereas another study reported a rate of 23% and identified it as second most commonly endorsed item [62]. Both studies were quite similar (i.e., *N* > 200, survivors were in their late 30s, about 2 years from diagnosis, used single CaSUN-item), but Bender and colleagues [58] suggested that their findings may be explained by recruiting relatively few survivors who had received chemotherapy. In fact, different treatment modalities can have diverse effects on sexual functioning [1, 46], which could determine different sexual health-related questions and care needs, but effects of different treatment regimens have not been tested thoroughly in the identified studies. The only study that compared needs between survivors who had received different types of treatment reported that testicular cancer survivors with more intense treatments (i.e., polychemotherapy and surgery) had higher needs [57]. However, the categorized treatment groups were confounded by survivors’ disease severity and more research is needed. Finally, impaired health status and fatigue were related to increased support needs [24, 59, 61], which highlights that sexual health-related needs should not be neglected in sicker survivors. Providers, survivors themselves, and maybe even partners/spouses should be cautious, as survivors with more health problems may have more specific needs surrounding sex-related questions and support.

Qualitative studies offered valuable details about the types of sexual health-related care needs, but over-represent female patients/survivors (*n* = 9/16 studies). Young adult patients/survivors highlighted that sexual health remains under-addressed in clinical practice. They described a silence around the topic of sex, or discussions with providers were inadequate, resulting in unmet needs. If oncology care providers do not address and normalize sexual health, patients/survivors may interpret their questions or concerns as irrelevant, further promoting a neglect of sexual health. A need for more detailed sex-related information was frequently identified, but the content of such needed information was rarely assessed or defined, which should be considered in greater detail in future research. Some studies identified needs for information about how to cope with physical side effects of cancer, with menopause, or with vaginal dryness. There appeared to be an overlap concerning such topics, where some studies indicated a need for support and others a need for more information. We suggest that both can go hand in hand and the provision of information in itself may also be a form of support. Support and information needs partly differ by relationship status, as singles voiced other questions and related needs than partnered patients/survivors (e.g., disclosure and dating new partners vs. communicating sex-related changes with current partners). Other insightful comments about information needs included that providers mentioned various treatment-related effects on the body, but did not discuss any impact on or implications for sexual health. Such impact may not be readily apparent for patients, leaving them ill-prepared for potential side effects of their cancer treatment. Providing more detailed information can help patients/survivors to anticipate and adjust to potential changes in their sex lives. However, it remains to be tested what kind of information which patients and survivors need. Importantly, the way providers communicate such information is vital, as they should initiate conversations and be open. Discussing sexual health can easily be included in the discussion on any side or long-term effects of cancer treatment. Thereby, providers should also have sufficient knowledge of (online) resources or specialists in their area to further refer patients/survivors if needed (Table 2).

Other points of consideration, based on this review, include that some studies combined needs related to sexual health with fertility [73, 75, 80, 83] or they identified low importance of counseling about sexual health, but highest importance for fertility-related questions [68]. We want to highlight that conversations about fertility may easily be used to also open conversations about sex and sexual health. At the same time, we urge providers and researchers to not over-emphasize fertility while neglecting sexual health of young adults during and following cancer treatment. Providers should also be mindful of differences in cultural or personal background, given that sex and sexual health can be topics filled with shame and embarrassment. We advocate for providers to respect the boundaries of patients/survivors and not force any conversation, but still make patients/survivors aware of potential problems and available support. Several providers could be tasked with providing sex-related information and support, where repetition of available resources and knowledge of whom to contact can be key for patients and survivors. Interestingly, survivors and oncology care providers seem to differ in their views on which provider is responsible for sex-related discussions [86], with survivors indicating nurses or sexologists, whereas providers indicated physicians and nurses as responsible. It remains crucial for future studies to further delineate which support and information patients/survivors desire to be able to anticipate who should take responsibility in a multidisciplinary clinical care team.

This review’s thorough search, excellent inter-rater reliability, and clear focus represent definite strengths, but some limitations should be considered. We focused on care needs that should be addressed by providers, whereas patients and survivors are certainly also in need of informal support from peers, partners/spouses, or their extended family. Professional help may not always be indicated, but providers can, for example, still help guide the way to information about peer/patient support groups. Moreover, our risk of bias assessment of all included studies could not be used to thoroughly guide the interpretation or relevance of findings, given various approaches to addressing sexual health and diverse groups of participants in the included studies. Nevertheless, this overview may further help guide readers in determining the relevance of each study for their own purpose (Online Resource 3).

In sum, this review highlights that support needs regarding sexual health exist and vary among patients and survivors diagnosed with cancer during young adulthood. Patients and survivors called for additional age-appropriate practical/emotional support and information. The specific nature of such support and content of needed information needs more detailed assessment in future research, but identified examples refer to support/information on physical side effects, dating/communicating with romantic partners, and coping with sexual difficulties. Ways of how sex-related information should be communicated by providers were clearly described and include initiating sexual health-related discussions that ought to be open, empathetic, and should validate patents’/survivors’ concerns. Such conversations could be face-to-face, or providers could guide patients/survivors to appropriate online resources.

There has been a call to improve sex-related communication in oncology for years [37–40], but the translation into clinical practice seems suboptimal given that patients, and survivors still highlight inadequate care. There seems to be a disconnection between advocating and implementing adequate care, which may lay in how to identify and tackle problems in the clinical setting. This review offers an overview of needs that partly remain generic/broad, but also underlines the importance of focusing on sexual health and related needs in young adult cancer care. For now, an individualized approach of trying to understand individual patients’/survivors’ questions, concerns, and associated needs is recommended to offer tailored help, where empathetic and open conversations seem key. Thereby, the communication principles of the extended PLISSIT [89] or BETTER [90] models may help providers to consciously open and allow for sex-related communication with patients and survivors [46]. Finally, we also encourage clinicians and researchers to assess unmet needs by different sexual health domains (e.g., interest vs. arousal vs. functioning). This can offer more detailed revenues to better tailor sexual health-related care to the needs of different patients/survivors of young adult cancer in the future.

## Supplementary Information

Below is the link to the electronic supplementary material.

Supplementary file1 (PDF 359 KB)

## Data Availability

See Online Resources 1-3.

## Declarations

### Disclaimer

The views expressed in this article are the author’s own and not an official position of the funder or their affiliations.
