# Supplementary material for: Sexual health-related care needs among young adult cancer patients and survivors: a systematic literature review
Source: J Cancer Surviv. 2021 Oct 20;16(4):913–24. doi: 10.1007/s11764-021-01084-w (PMC9300501; doi:10.1007/s11764-021-01084-w)
Supplement: Supplementary file 1 — Supplementary file1 (PDF 359 KB) [file 11764_2021_1084_MOESM1_ESM.pdf]

Appendix to the manuscript:

Sexual Health-Related Care Needs among Young Adult Cancer Patients and Survivors: A Systematic Literature Review

Lehmann, Laan, & Den Oudsten

Online Resource 1:
Database search strategy *page 1*
Online Resource 2a:
Overview of study characteristics and outcomes of quantitative survey-based studies (*n*=19) *page 2*
Online Resource 2b:
Overview of study characteristics and outcomes of qualitative/ mixed method studies (*n*=16) *page 6*
Online Resource 3:
Risk of bias indicators of all studies (*N*=35) *page 11*

Online Resource 1: Database search strategy

	Topic	Search terms	Notes
**1**	cancer	cancer OR<br>neoplasm OR<br>malignancy OR<br>oncology OR<br>tumor	Search field: Abstract<br>(to eliminate papers that only mention relevant terms in the introduction, discussion, affiliations; or references)
**2**	sex	sexual OR<br>"sex-related" OR<br>sexuality OR<br>"sexually active" OR<br>"sexually inactive" OR<br>"sexually experienced" OR<br>"sexually-related" OR<br><br>"supportive care needs" OR<br>"supportive health care"	Search field: Abstract<br>- we did not use "sex" to eliminate papers that only refer to biological sex (male/female) as sample descriptor<br>- we did not use "sexually" / "sexu*" to eliminate papers on "sexually transmitted/ abused/ mature …" instead we added specific terms like "sexually active/ experienced"<br><br>- we also included "supportive /heath care needs" here, because sexual health-related needs are sometimes subsumed under this broader term
**3**	needs	need OR<br>needs OR<br>"supportive care" OR<br>support OR<br>care OR<br>concerns OR<br>problems OR<br>barriers OR<br>difficulties	Search field: Abstract<br>- we did not use "need*" (but need OR needs) to prevent detecting many papers that used the verb 'needed' / 'needing'
**4**	age	"young adult*" OR<br>"AYA" OR<br>"age-specific" OR<br>younger	Search field: Abstract<br>- "young adult*" extends to adult<u>s</u> and adult<u>hood</u><br>- using "younger" also identified studies that did not specify "young adults" but compared subgroups (younger vs. older)
**5**		S1 AND S2 AND<br>S3 AND S4	[= combining all 4 topics]
**6**		childhood OR<br>pediatric OR<br>prostate OR<br>Papillomavirus	Search field: Title<br>[i.e., focus of the paper]
**7**		S5 <u>NOT</u> S6	= to exclude:<br>(a) studies on young adult survivors of childhood cancer [note: there are guidelines focusing on childhood, adolescent, and young adult survivors but they do not contain original data or focus on sexual health]<br>(b) studies on typically older prostate cancer patients<br>(c) HVP vaccine/cancer prevention studies (by using 'papillomavirus' and not 'HPV', we excluded vaccine-related papers, but kept those stating 'HPV-related cancers')<br>+ limited to 'Human studies'

Online Resource 2a: Overview of study characteristics and outcomes of all quantitative survey-based studies (n=19)

1st author<br>Year<br>Location	N<br>(RR)	Sample specifics	Type dx, (most common)	Age at/<br>time since dx	Survival	Age at study	Study focus<br>Results related to sexual health-related care needs
Aggarwal<br>2020<br><br>Canada<br>[87]	129<br>(RR=54%)	52% female	mixed, genitourinary	M_{time}=2.9y (4.7)<br>Md_{time}=1.4y	Short to mid-term survival	30<br>(6.1)<br>15-39	**Study focus**: Health-related social media use<br>**Results:**<br>- Seeking online information about sexual health was highest among 25-29 year-olds (29% vs. 9% among 20-24 year-olds)<br>- Desired sexual information in a resource to help with cancer care most often endorsed by 30-34 year-olds (35% vs. 12% among 20-24 year-olds)<br>- Relative to older survivors (40-65 years, *n*=157), AYAs regard information about sexual health more relevant for future online resources (4% vs. 24%)
	**Method**: quantitative;<br>self-developed survey items, incl. "Which information topics are you looking for in a resource to help with your cancer care?" [sexual health]						
Albers<br>2020<br><br>Netherlands<br>[86]	56<br>(RR=39%)<br>52 provider<br>(RR=29%)	79% female	mixed, hematological	M_{age}=26 (5.2),<br>15-36<br>Time: 3m – 10+y	Across all ranges of survival	29<br>(5.0)<br>20-41	**Study focus**: Communication preferences about sexuality and intimacy<br>**Results:**<br>- 27% reported providers sufficiently discussed sexuality<br>- Best way to provide information: online (66%), face-to-face (64%)<br>- AYAs: NPs (62%) or sexologist (49.1%) are most suitable for sex-related discussions; providers view physicians (84%) or NPs (84%) as responsible<br>- Reasons for *not* talking about sex: provider did not initiate conversation (18%), lack of privacy (16%), provider had opposite sex (13%); provider is too busy (8%), provider does not seem open (5%)
	**Method**: quantitative;<br>self-developed survey items, e.g., "Did you ever receive information from a health care professional about the effects of the disease and treatment on sexuality?; What could be improved about the information on intimacy and sexuality?"						
Bender<br>2012<br><br>Canada<br>[58]	204<br>(RR=71%)	100% male	testicular	M_{time}=2.2y (1.1)	Mid-term survival	36<br>(10.5)	**Study focus**: Supportive care needs and use of online support<br>**Results:**<br>- 37% reported having sexuality-related needs<br>- 48.2% reported needs in adjusting to how they felt about their bodies<br>- Sex-related health needs not included in top-10 met or top 10-unmet needs
	**Method:** quantitative;<br>survey, including an 'adapted' version of the CaSUN questionnaire [55] [modifications not specified]						
Dyson<br>2012<br><br>Australia<br>[65]	53<br>(RR=74%)	43% female	mixed, sarcoma	M_{time}=40 days,<br><4m post dx	Short-term	21<br>(0.5)<br>16-30	**Study focus:** Relationship between unmet needs and distress<br>**Results:**<br>- Sexuality need items were <u>not</u> reported as one of the top-20 unmet needs [out of 34 items]<br>- Sexuality needs were unrelated to state anxiety or depression
	**Method**: quantitative;<br>survey, including the SCNS-SF34 subscale: sexuality [54]						
Geue<br>2015<br><br>Germany<br>[59]	99 /146<br>(from existing data)	66% female, partnered	mixed, hematological	Age: 15-39<br>M_{time}=30 m	Across all ranges of survival	33<br>(5.6)<br>18-45	**Study focus:** Sexuality and romantic relationships<br>**Results:**<br>- Highest need for support item: changes in sexual feelings (*n*=38, 38.3%)<br>- Sexuality needs domain: *M*=24.1 (29.9)<br>- Women reported higher sexuality needs than men: 28.5 vs. 15.2, [d=0.45]<br>- Fatigue associated with greater sexuality needs; and high sexual satisfaction associated with decreased sexuality needs
	**Method**: quantitative;<br>survey, including the SCNS-SF34 subscale: sexuality [54]						

<table>
<tr><th>1st author
Year
Location</th><th>N
(RR)</th><th>Sample specifics</th><th>Type dx, (most common)</th><th>Age at/
time since dx</th><th>Survival</th><th>Age at study</th><th>Study focus
Results related to sexual health-related care needs</th></tr>
<tr><td rowspan="2">Graugaard
2018

Denmark
[34]</td><td>822
(RR=45%)</td><td>51% female, national survey</td><td>mixed, melanoma</td><td>M_{age}=24, 15-29
(18% <19 at dx)
M_{time}=3.9, 1-7y</td><td>Across all ranges of survival</td><td>28,
17-36</td><td rowspan="2">Study focus: Sexual and romantic challenges
Results:
- Occurrence of sex-related conversations <u>during hospitalization</u>:
49.5% little/no discussion (=unmet need); 18.0% reported no need
- Needs were higher in genital (OR=20.5) and breast (OR=24.4) cancer survivors (relative to melanoma), but unmet needs did not differ. Unmet needs were 2 times more common among female than male survivors
- Occurrence of sex-related discussions <u>during follow-up</u>:
61.7% unmet, 17.3% no need
- Needs were higher in genital (OR=8.1) and breast (OR=15.2) cancer survivors (relative to melanoma) and those with sexual problems in the past week (OR=2.2), but unmet needs did not differ. Unmet needs during follow-up were 1.7 times more common among female survivors</td></tr>
<tr><td colspan="6">Method: quantitative;
Self-developed survey about body image, sexuality, and romantic capability, including 2 questions: “Has a professional at the hospital talked with you about how your illness or treatment might affect your love life or sex life?” and “During follow-up consultations, did a doctor or a nurse talk with you about whether you had problems in relation to being in love, partners or sex?” [answers: to a lesser extent / not at all = unmet need]</td></tr>
<tr><td rowspan="2">Gupta
2013

Canada
[68]</td><td>243
(RR=96%)</td><td>61% female</td><td>Mixed, lymphoma</td><td>40% on tx,
max. 5 years post dx</td><td>Very short to mid-term survival</td><td>28,
17-35</td><td rowspan="2">Study focus: Information and service needs
Results:
- Importance of counseling related to sexuality or intimacy during treatment: median=7, M=6.3, range:1-10 [appeared to be on the lower end of information and service needs] highest: information about healthy life style or fertility
- Ratings did not differ by sex, but those living with a partner reported higher interest in sex/intimacy counseling than YAs living with parents or roommates</td></tr>
<tr><td colspan="6">Method: quantitative;
Survey, including an age-specific needs questionnaire [24]; accompanied by an importance rating</td></tr>
<tr><td rowspan="2">Hall
2012

Australia
[60]</td><td>58
58 OAs (existing data)</td><td>71% female</td><td>Mixed, melanoma</td><td>M_{age}=35, 18-40
(M_{age}=70, 40-85)
M_{time}=7 months</td><td>Short-term survival</td><td>18-40</td><td rowspan="2">Study focus: Quality of life, unmet needs, and health behaviors
Results:
- Unmet sexuality needs:
29.3% among YAs vs. 10.7% among sex- and cancer type-matched older adult (OA) survivors age 40-85 years (p=.001)</td></tr>
<tr><td colspan="6">Method: quantitative;
survey, including the SCNS-SF34 subscale: sexuality [54]</td></tr>
<tr><td rowspan="2">Jonker Pool
2004

Netherlands
[57]</td><td>264
(RR=85%)
50
(RR=73%)</td><td>100% male</td><td>Testicular, lymphoma</td><td>M_{age}=29 / 34
(17-70)
M_{time}=5.9y</td><td>Long-term survival</td><td>36 /
42</td><td rowspan="2">Study focus: Male sexuality after cancer: information and support needs
Results:
- Need for information about sexuality:
67% testicular vs. 27% lymphoma survivors
- Need for support regarding sexuality:
21.5% testicular vs. 8% lymphoma survivors
- Among testicular survivors: need was highest among those treated with combined polychemotherapy and surgery relative to other treatments</td></tr>
<tr><td colspan="6">Method: quantitative;
Self-developed survey items; including two questions that assess the current need for information and support (yes/no)</td></tr>
<tr><td rowspan="2">Kedde
2013

Netherlands
[56]</td><td>332 [c]</td><td>100% female</td><td>breast</td><td><6 y post dx;
43% completed tx</td><td>Very short to mid-term survival</td><td>39,
22-49</td><td rowspan="2">Study focus: Sexual dysfunction
Results:
- 52% were consulted on potential changes in sexuality (typically by a nurse), and 87% were satisfied with provided information
- 50% of those with sexual dysfunction (n=186) ever perceived a need for sex-related healthcare, but 60% did not receive it [= 25% of total sample ever perceived a need, which remained unmet in 15.2% of total sample]</td></tr>
<tr><td colspan="6">Method: quantitative;
Self-developed survey items, assessing the ‘use and need of care’ : “whether the subject of changes on sexuality had ever been brought up” and by whom</td></tr>
</table>

1st author<br>Year<br>Location	N<br>(RR)	Sample specifics	Type dx, (most common)	Age at/ time since dx	Survival	Age at study	Study focus<br>Results related to sexual health-related care needs
McCarthy<br>2018<br><br>Australia<br>[85]	196<br>(RR=26%)	49% female	Mixed, leukemia	M_{age}=20 (3.2),<br>15-25;<br>Time: 6-24m;<br>81% completed tx	Short-term survival	22,<br>15-27	**Study focus:** Information needs of patients and parent-carers<br>**Results:**<br>- 1/3 reported (any) unmet information need (highest need: long-term effects on health: 51%)<br>- Information on sex/sexuality in relation to cancer and treatment:<br>- Total need: 87% (*n*=167/193), of which 31% reported unmet needs (*n*=52/167); 13% indicated: does not apply (*n*=26/193)<br>- Ranked #13 out of 17 assessed items
	**Method**: quantitative;<br>Adapted 17-item information needs survey (based on 13-item AYA HOPE survey [91]), incl. 1 information needs item: “sex/ sexuality in relation to cancer treatment”						
Mutsch<br>2019<br><br>Germany<br>[69]	577<br>(RR=76%)	74% female	Mixed; breast	M_{age}=29 (6.1),<br>18-39<br>M_{time}=11.9m (8.0),<br>83% completed tx	Short-term survival	nr	**Study focus:** Sexuality and cancer: Reproductive organ cancers vs. others<br>**Results:**<br>- Women (*n*=424) reported higher sexuality needs than men (*n*=153)<br>- Among female survivors: those with reproductive organ cancers (*n*=201) reported higher sexual health-related care needs than others (*n*=223; *d*=0.26)<br>- Among male: no differences in sexual health-related care needs between those with reproductive organ cancers (*n*=50) vs. others (*n*=103; *d*=0.05)
	**Method**: quantitative;<br>survey, including the SCNS-SF34 subscale: sexuality [54]						
Park<br>2011<br><br>Korea<br>[66]	179/1084<br>(group IV)<br>(RR=87%*)	100% female	breast	min. 5y post-surgery	Long-term survival	44	**Study focus:** Unmet needs relative to survival duration<br>**Results:**<br>- Sex-related items were not included in the top-10 most reported need items<br>- Sexuality subscale score: adjusted *M*=1.83 (1.0) were similar to survivors who were older and closer to surgery (p>.1)
	**Method**: quantitative;<br>survey, including the SCNS-LF59 subscale: sexuality [53]						
Sender<br>2019<br><br>Germany<br>[61]	514<br>(at T_2; 11% drop-out	75% female	Mixed, breast	M_{age}=30 (6.1),<br>18-39<br>M_{time}=12.1m<br>(<4y at T_1)	Short- to mid-term survival	nr	**Study focus:** Unmet supportive care needs and changes over time<br>**Results:**<br>- 49.6% reported at least 1 sex-related unmet need at T_1, and 46.9% at T_2 [= 2nd lowest rank of 6 supportive care domains]<br>- Needs for sex-related support did not change over time (*M*=28 vs. 26, *d*=.007) [note: scores were converted to a 0-100 scale]<br>- Lower self-reported illness adjustment was related to higher sexuality needs
	**Method**: quantitative;<br>survey, including the SCNS-SF34 subscale: sexuality [54]						
Sender<br>2020<br><br>Germany<br>[67]	117<br>(RR: nr)	66% female	Mixed, hematological	M_{time}=29.3m,<br>50% <2 y post dx	Short- to mid-term survival	31,<br>18-39	**Study focus:** Distress, supportive care needs, and satisfaction with care<br>**Results:**<br>- Sexuality needs domain rated 3rd highest of 5 domains (*M*=23.0) [note: scores were converted to a 0-100 scale]
	**Method**: quantitative;<br>survey, including the SCNS-SF34 subscale: sexuality [54]						
Smith<br>2013<br><br>Australia<br>[62]	244<br>(RR=70%)	100% male	testicular	M_{age}=35, 16-69<br>M_{time}=2.3y (<5) post tx	Mid-term	38<br>(10.3)<br>21-68	**Study focus:** Prevalence and correlates of supportive care needs<br>**Results:**<br>- 2nd most common unmet need: help to address my/our sex life (23%)
	**Method**: quantitative;<br>survey, incl. 1 CaSUN item of the relationship subscale [55]						

1st author<br>Year<br>Location	N<br>(RR)	Sample specifics	Type dx, (most common)	Age at/<br>time since dx	Survival	Age at study	Study focus<br>Results related to sexual health-related care needs
Zebrack<br>2009<br><br>USA<br>[24]	879 [c]	72%<br>female	mixed,<br>Hodgkin	M_{age}=26 (5.8),<br>15-35<br>M_{time}=4.7y	Across all ranges of survival	30<br>(8.1)<br>18-39	**Study focus:** Information and service needs<br>**Results:**<br>- 40.2% needed counseling/ guidance related to sexuality and intimacy, of which 73.7% expressed it as remaining unmet [=30% of total sample]<br>- Unmet needs for sex/intimacy counseling were more common among: female, unemployed, non-white survivors, and/or those with decreased health status<br>- unmet need among female: 33.1% vs. male: 21.4%
	**Method**: quantitative;<br>Self-developed needs-specific questionnaire (which was used in later studies, also included in this review, i.e. [63,68]						
Zebrack<br>2013<br><br>USA<br>[63]	111/ 215<br>(RR=75%)	53%<br>female	mixed,<br>breast	age:<br>41%:20-29y<br>59%:30-39y<br>M_{time}=66 days	Very short term survival	31<br>(6.0)	**Study focus:** Psychosocial service use and unmet needs<br>**Results:**<br>- counseling or guidance related to sexuality or intimacy:<br>- age group 20-29:<br>14.6% unmet need (2.1% used service, 83.3% reported no need)<br>- age group 30-39:<br>21.5% unmet need (4.6% used service, 73.8% reported no need)
	**Method**: quantitative;<br>Survey, including needs-specific questionnaire [24]						
Zhou<br>2017<br><br>China<br>[88]	56/ 173<br>(RR=89%)	100%<br>female	cervical	50% completed tx in year prior to study	Short-term	19-45	**Study focus:** Patterns and predictors of healthcare-seeking for sexual problems<br>**Results:**<br>- 2.9% of total sample (n=5/173) received help regarding sexuality<br>- YA patients reported interest in sex-related counseling more often (75% vs. 43% among older survivors), but 63% of YA were not ready to seek help<br>- Most common reasons (among full sample): embarrassment, prejudice/stigma
	**Method**: quantitative;<br>nurse-led face-to-face interview, including a self-developed survey to assess: interest in receiving care to address sexual issues, likelihood of seeking such care, history of seeking care for sexual health problems						

YA = young adult; AYA = adolescent and young adult
SCNS-SF34 = Supportive Care Needs Survey, short-form (incl. 34 items [54]); SCNS-LF59 = long-form (incl. 59 items [53]), both version include the 3-item sexuality subscale
CaSUN=Cancer Survivors' Unmet Needs measure [53], which includes the relationship subscale that has 1 item referring to 'sex life'
c = convenience sample (i.e., response rate cannot be determined)
nr = not reported
? = could not (reliably) extrapolated from reported data
* 87% = completion rate (i.e., refers to the percentage of consenting participants that completed the survey)

Online Resource 2b: Overview of study characteristics and outcomes of all qualitative/ mixed method studies (*n*=16)

1st author<br>Year<br>Location	N<br>(RR)	Sample specifics	Type dx, (most common)	Age at/ time since dx	Survival	Age at study	Study focus<br>Results related to sexual health-related care needs
Bolte<br><br>2010<br><br>USA/ Canada<br>[72]	8/113	38% female (=3/8)	mixed,<br>8 different dx	M_{age}=27<br>7/8 in remission	Mid-term survival	30,<br>21-38	**Study focus**: Impact of cancer on the sexual self<br>**Results:**<br>- Theme #6/6: *Providers' insensitivity to sexual issues highlights the need for improved communication*:<br>- late effects were mentioned, but implications for sex were not addressed<br>- Providers would not have discussed sex unless participants brought it up<br>- Sex-related needs and concerns were dismissed/ not validated; providers did not show empathy<br>- lack of communication, support resources, or validation led to feelings of isolation and lacking trust in providers (n=1/8 received information that validated and normalized physical and emotional changes related to sex)<br>- lack of information was perceived as sexual health concerns/ needs being unimportant
	**Method**: qualitative;<br>One phone focus group (of randomly selected participants of a survey among *N*=113), guided by McGill Illness Narrative (MINI) semi-structured interview protocol, adapted to include 'sex life'						
Connell<br>2006<br><br>Australia<br>[64]	35 [c]	100% female	breast	M_{age}=35 (20-40),<br><4y post dx	Short to mid-term survival	Md: 35,<br>23-43	**Study focus**: Issues and concerns of young women with breast cancer<br>**Results:**<br>- 3% reported unmet needs related to 'sexual issues' [not further described]<br>- Most common unmet needs: emotional and physical support (37%); information needs and health service concerns (25.5%)<br>- 28.5% received support that was not age-appropriate
	**Method:** qualitative [incl. quantitative reports of frequencies];<br>Semi-structured home or phone interviews following a question guide with open-ended questions [which did not specify sex-related issues]						
Corney<br>1993<br><br>UK<br>[70]	33/105<br>(RR=76%)	100% female	gynecological	6m - 5y<br>post-surgery	Short to mid-term survival	<40,<br>27-95	**Study focus:** Psychosexual dysfunction<br>**Results:**<br>- Out of all 105 women, *n*=29 spontaneously mentioned they needed more sex-related information [not further described]. It was uttered by 50% of women under age 40 (i.e., n~16/33) vs. 8% of women above age 50
	**Method**: qualitative;<br>Semi-structured interviews on psychosocial/psychosexual adjustment						
Dobinson<br>2016<br><br>USA<br>[73]	11 [c]	55% female	mixed, breast, testicular	M_{age}=28 (8.5),<br>15-45	Across all ranges of survival	32<br>(7.5)<br>21-43	**Study focus:** Psychosexual unmet needs<br>**Results:**<br>- 82% (*n*=9/11) reported at least 1 unmet psychosexual need<br>- Identified 6 areas of psychosexual unmet needs:<br>- Fertility (need for age-appropriate information, support in coping with infertility)<br>- Sexual communication (learn how to assert oneself sexually and to discuss sex openly with partners)<br>- Cope with physical side effects (e.g., menopause, vaginal dryness)<br>- Dating and disclosure (assistance in re-entering the dating scene)<br>- Desire to connect with other AYAs<br>- Reconciling identity conflict (i.e., resolve friction between actual vs. perceived age; need for information about sexual milestones from providers)<br>- Most sought support themselves, but needed more (face-to-face or online)<br>- Factors contributing to psychosexual needs being unmet:<br>- physician neglecting sexual wellbeing due to incorrect assumptions (*n*=7/11)
	**Method**: qualitative;<br>semi-structured interviews, including topics based on previous literature (i.e., impact of cancer on sexual function, relationships, and sexuality)<br>constant comparison approach						

1st author<br>Year<br>Location	N<br>(RR)	Sample specifics	Type dx, (most common)	Age at/ time since dx	Survival	Age at study	Study focus<br>Results related to sexual health-related care needs
							- participants neglect sexual well-being during treatment (n=4/11), but desire more information<br>- Psychosexual concerns may differ by age and developmental stage; and were more common among survivors of sexual organ-related cancers [not tested statistically given study design and N=11]<br>- Participants emphasized including partners if possible
Gorman<br>2020<br><br>USA<br>[74]	29 [c]<br>25 male partners	100% female	breast	M_{age}=31 (24-39);<br>3-4y post dx	Mid-term survival	36,<br>29-42	**Study focus:** Sexual health in cancer survivorship: Dyadic perspective<br>**Results:**<br>- Theme #5/5: need for services and support:<br>- Lacking help to manage sexual difficulties, especially for partners/ couples<br>- Preferences of receiving services/ support:<br>couples may be worried about stigma, but couples' therapy was most commonly suggested approach; 50% also preferred an online format; there is "no one size fits all solution" and support could be provided based on personal preferences (e.g., online, counseling, couples' therapy)<br>- Note: theme #2/5: Dyadic management of sexual health (e.g., navigating changes; getting through it as a couple/team)
	**Method**: qualitative;<br>semi-structured phone interviews (separate for survivors and partners), interview guide was based on literature and feedback from experts; including questions about the couple's relationship, sexual health, communication, and perspectives on their sexual relationship						
Gould<br>2005<br><br>Canada<br>[75]	65 [c]	100% female	breast	M_{age}=37 (26-45)	?	41,<br>26-51	**Study focus:** Information and support experiences, and needs among young women with breast cancer<br>**Results:**<br>- Overall theme: "Nothing fit me", including: 'Lack of information':<br>- No answers to their questions, e.g., about treatment effects on the body (e.g., fertility problems, early menopause), how to relate to themselves sexually following treatment, how to contemplate dating, how to talk about their cancer in new relationships<br>- If generic information was available, it was inappropriate/ not age-specific<br>- Participants described a "silence surrounding sexual dysfunction, how to understand the sexual changes to their bodies and how to feel sexual again with others"; how to start new relationships was relevant for singles<br>- Participants' suggestion to address lack of information: oncology care providers (in clinic or community-based) should create and implement topical workshops (e.g., about dating, sexuality, recurrence, finances, nutrition)
	**Method:** qualitative;<br>10 focus groups across Canada, which were stratified by whether women were single/partnered and with/without children						
Lewis<br>2012<br><br>USA<br>[76]	33<br>(RR=69%)	100% female, African American	breast	M_{age}=37 (6.0)	?	25-45	**Study focus:** Psychosocial concerns<br>**Results:**<br>- 73% received no sex-related information from providers; 9% received minimal information; 18% recalled a reasonable discussion of sexuality<br>- 52% wanted more sex-related information<br>- Taboos about discussing sexuality in the African American community can exacerbate poor communication (neither providers nor patients address it)<br>- 27% had used some type of treatment for a sexual problem (i.e., lubricants, estrogen replacement, vaginal dilators, used herbal remedies)
	**Method**: mixed;<br>Semi-structured interview of 49 questions, incl. 3 about sex: "*How much did your medical team tell you about breast cancer and sexuality? Was the information you received not enough, about right, more than you wanted? Have you used any kind of treatment for a sexual problem?*"						

1st author<br>Year<br>Location	N<br>(RR)	Sample specifics	Type dx, (most common)	Age at/<br>time since dx	Survival	Age at study	Study focus<br>Results related to sexual health-related care needs
Lopez<br>2019<br><br>Australia<br>[71]	19 'young'<br>19 'old'<br>(RR: nr)	100% female	gynecological	Age 'young': 27-49 (16/19 were <39)<br>Age 'old': 50-70;<br>Time: 1m – 5y post tx	Short to mid-term	27-70	**Study focus:** Age-related supportive care needs<br>**Results:**<br>- Theme 5/8: *Asking questions is hard*:<br>- Some younger women needed more information about (coping with) sexual difficulties, but found it difficult to initiate conversations with providers, especially with male providers<br>- Theme 6/8: *Loss*<br>- sexual functioning was important to all women, but younger women reported unmet needs more often (e.g., related to sexual difficulties during treatment, or forming a barrier to establishing future relationships)<br>- treatment-related physical changes caused alienation from their own bodies, resulting in unmet needs regarding body image
	**Method**: qualitative;<br>semi-structured phone interviews with questions based on previous literature; qualitative thematic analyses to establish categories of (un)met needs						
Mattson<br>2018<br><br>Sweden<br>[77]	337<br>(RR=52%)	100% female	gynecological	M_{age}=33 (4.9), 19-39<br>M_{time}=2.9y (1.9)	Short- to mid-term survival	37 (5.1)	**Study focus:** Perceived distress and provider support following treatment<br>**Results:**<br>- 85% reported cancer-related concerns; and based on open-ended questions: 30.4% specified concerns about sexuality (=5th most common concern)<br>- Of women who needed and sought support (*n*=159), 34% felt the provided support was inadequate (*n*=55/159):<br>- Support was not useful or tailored to patients' problems/ needs; providers were uncomfortable (e.g., talking about masturbation)<br>- Reasons for <u>not</u> seeking support: problems were too private/intimate; survivors did not know where to find support (e.g., sexologist)
	**Method:** mixed;<br>Self-developed closed and open-ended survey questions, for example : "Have you experienced a need for support?" (yes/no); "Did you receive the support you needed?" (yes/no), "If you did not receive the support you sought, please describe the reasons why" (open response)						
Miedema<br>2013<br><br>Canada<br>[78]	53 [c]	74% female	Mixed, breast	M_{age}=29 (5.9), 18-39<br>Time: <1-5+ y	Across all ranges of survival	32 (6.4) 20-44	**Study focus:** Follow-up care meeting survivors needs?<br>**Results:**<br>- overarching theme: age-specific care is needed<br>- 1 out of 4 subthemes: psychological care needs: psychological care was needed given the absence or inadequacy of services (incl. family, couples, and sexuality counseling)
	**Method**: qualitative;<br>semi-structured phone interviews using 11 open-ended questions, constructivist grounded theory framework						
Olsson<br>2015<br><br>Sweden<br>[84]	23/44<br>(RR=33%)	sex: nr by subgroup	mixed, (nr by subgroup)	Age: 19-29<br>Time: 3-48 months since tx	Short-term	19-31	**Study focus:** Experiences of cancer care<br>**Results:**<br>- Identified 4 generic categories: (1) personal professional interaction, (2) knowledge and participation, (3) age-appropriate environment, (4) support<br>- Theme #2: subcategory: knowledge of sexuality and fertility:<br>- Sought additional information<br>- Lacked information, expressed need for increased discussion<br>- Expected providers to raise the subject
	**Method:** qualitative;<br>6 in-person focus groups (incl. 2-7 participants each), starting with open questions about care needs about personal experiences and importance; followed by addressing different types of care: *Environment, Decision-making, Family/significant other, Respect and integrity, Communication, Close relationships, Competence (of providers), Continuity*						

1st author<br>Year<br>Location	N<br>(RR)	Sample specifics	Type dx, (most common)	Age at/<br>time since dx	Survival	Age at study	Study focus<br>Results related to sexual health-related care needs
Ruddy<br>2013<br><br>USA<br>[79]	36<br>(RR: nr)	100% female	breast	Age: 18-42<br>M_{time}=22m (3-53)	Short to mid-term survival	38<br>(4.7)<br>26-44	**Study focus:** Unmet needs<br>**Results:**<br>- Major themes: (1) feeling different, (2) facing unique challenges, (3) desiring assistance<br>- Theme #1.3: survivors experienced physical changes and distress, and complained that sexuality was barely discussed<br>- Theme #3.3: suggestions/ need for additional education materials were expressed (e.g., pamphlets about any side effects, including sexual function)
	**Method:** qualitative;<br>4 in-person focus groups, hosted by a trained moderator who utilized a semi-structured interview guide (based on previous literature); questions aimed at experiences across the whole disease continuum						
Ruddy 2015<br><br>USA/ Canada<br>[80]	20 [c]	100% female; 50% non-White	breast	Age: 26-42;<br>Time: < 4y	Short to mid-term survival	36<br>(5.0)<br>26-42	**Study focus:** Support and unmet needs<br>**Results:**<br>- 10 themes emerged: 3 existing/met supports, 5 issues with inadequate information, and 2 additional unmet needs<br>- needs did not seemingly differ by race/ ethnicity<br>- "*Needed information and support*" :"*Menopause, sexual function, and fertility*"<br>- Early menopause and changes in sexual functioning not addressed adequately, women desired additional information about increasing libido and reducing vaginal dryness<br>- Due to lack of information from providers, some women turned to other sources of support (friends, internet)<br>- "*Guidance on navigating current and future romantic relationships*":<br>- Need more information about how a cancer diagnosis may negatively affect intimate relationships.<br>- Single women uttered concern and need for support dating new partners
	**Method:** qualitative;<br>semi-structured phone interviews, including 5 core open-ended questions (based on 'an ecological model' and) designed to explore existing support and unmet needs for information and support						
Thompson<br>2003<br><br>Australia<br>[81]	8<br>(RR=16%)	38% female	mixed,<br>8 different dx	M_{age}=24 (22-29)	nr	22-30	**Study focus:** Issues in transition from active to follow-up care<br>**Results:**<br>- 3 major categories were identified: information provision, treatment process and post-treatment care/survivorship<br>- *Treatment process* highlighted a "lack of skilled and timely interventions", incl. the sub-category '*Sexuality & Body Image*' [not defined or further described]
	**Method:** qualitative;<br>One supervised focus group; data were analyses using a grounded theory approach						
Tsangaris<br>2014<br><br>Canada<br>[82]	8/ 20<br>(RR=67%)	sex: nr by subgroup	mixed,<br>(nr per subgroup)	Age: 19-25	nr	19-25	**Study focus:** Identifying supportive care needs<br>**Results:**<br>- 2 out of 7 identified need-related themes included sex-related subcategories<br>- Theme*: Need for information-sharing and communication*,<br>- subcategory: Sexual function (lack/ need for information to be prepared)<br>- Theme: *Need for service provision*<br>- subcategory: needing time alone with doctor to discuss sensitive matters<br>- subcategory: doctors need to be prepared to talk about sexual health<br>- Qualitative findings echoed findings from the literature review (incl. needs related to information sharing and service provision)
	**Method**: mixed;<br>Qualitative phone interviews and integrating findings from a literature review; Phone interviews with n=8 YAs and n=12 diagnosed in adolescence, using open-ended questions based on the Supportive Care Needs Framework (i.e., 7 domains of supportive care: physical, social, emotional, informational, psychological, spiritual, and practical)						

1st author<br>Year<br>Location	N<br>(RR)	Sample specifics	Type dx, (most common)	Age at/<br>time since dx	Survival	Age at study	Study focus<br>Results related to sexual health-related care needs
Zebrack<br>2006<br><br>USA<br>[83]	37<br>(RR=79%)<br>40 providers<br>(RR=70%)	sex: nr	mixed, unclear	M_{age}≈23,<br>15 - 32	?	~31,<br>19-44	**Study focus:** Health care needs<br>**Results:**<br>- In round #1, four thematic categories were identified (incl. ~50 needs items), and the top-5 needs per domain were rated:<br>- The domain: *Counseling, Information, & supportive care needs for off-treatment survivors* included sex-related needs:<br>- Item which was ranked #2-3: Information about *sexuality, fertility, and reproductive risks/issues* (incl. pregnancy safety, option for having a family, menopausal symptoms, a genetic risks for offspring)
	**Method**: mixed;<br>Survey-based Delphi panel, including 3 iterative rounds:<br>Round 1: open-ended questions to identify biomedical and psychosocial needs of YAs with cancer;<br>Round 2&3: items were ranked to identify the top-5 needs per domain						

YA = young adult; AYA = adolescent and young adult
c = convenience sample (i.e., response rate cannot be determined)
nr = not reported
? = could not (reliably) extrapolated from reported data

Online Resource 3: Risk of bias indicators

An overview of potential **sampling**, **measurement**, or **reporting** bias in all *N*=35 studies is presented below; along with other positive and negative points of consideration.

Potential biases are highlighted in red and defined as:

Potential **sampling bias** if:

- *N*<50 (quantitative studies) or *N*<10 (qualitative studies)
- RR<50% or was not reported/ could not be calculated
- proportion of (fe)males >70% in studies that included participants of both sexes

Potential **measurement/ methodological bias** if:

- self-developed items are used (quantitative/mixed method studies)
  or only 1 focus was conducted (quantitative studies) as it questions data saturation

Potential **reporting bias** if:

- see description

Note:

Lighter shades of red were used for:

- other points of concern (e.g., modification of existing self-report questionnaires)
- convenience sampling (i.e., given that it in itself can introduce a selection bias, but it cannot be assessed)

[see below]

Risk of bias indicators

	N	Potential sampling bias RR	% female	Potential measurement bias	Potential reporting bias	Other points of consideration
**Quantitative studies**						
Aggarwal [87]	129	54%	52	Self-developed survey items		
Albers [86]	56	39%	79	Self-developed survey items		
Bender [58]	204	71%	male only	'modified' CaSUN [55] [modification not specified]	Sexuality only mentioned in Discussion/rank of need item not specified (not in top-10 met or unmet items)	
Dyson [65]	53	74%	43	SCNS-SF-34 [54]	Rank of sexuality need items not specified (i.e., not included in top-20 out of 34 items)	
Geue [59]	99	existing data	66	SCNS-SF-34 [54]		Focus: sexual health & needs<br>Partnered participants only
Graugaard [34]	822	45%	51	Self-developed survey items		Focus: sexual health & needs; National survey
Gupta [68]	243	96%	61	Needs Questionnaire [24] and self-developed importance rating	Needs itself were not reported, focus on ratings of importance of needs	
Hall [60]	58	existing data	71	SCNS-SF-34 [54]		Compared results to *n*=58 sex- and cancer type-matched older adults
JonkerPool [57]	264, 50	85%, 73%	male only	Self-developed survey items		Focus: sexual health & needs
Kedde [56]	332	c	female only	Self-developed survey items	Needs reported for those with sexual dysfunction: 60% did not receive support = 15.2% of total sample	Unclear if needs were only assessed in this subsample
McCarthy [85]	196	26%	49	Adapted AYA HOPE survey [91]		
Mutsch [69]	577	76%	74	SCNS-SF-34 [54]		
Park [66]	179 / 1084	unknown for subgroup*	female only	SCNS-LF-59 [53]	Reported RR=87% is completion not response rate; Rank of sexuality needs not specified (i.e. not in top-10); mean scores were adjusted for several factors (e.g., age, education, economic status, menopause, various treatment modalities)	Also recruited three other groups of survivors (*n* = 176 - 481) that were older and closer to diagnosis for comparison
Sender [61]	514	not reported	75	SCNS-SF-34 [54]		
Sender [67]	117	not reported	66	SCNS-SF-34 [54]		
Smith [62]	244	70%	male only	CaSUN [55]		
Zebrack [24]	879	c	72	Self-developed, later used as Needs Questionnaire [24]		
Zebrack [63]	111 / 215	unknown for subgroup*	55	Needs Questionnaire [24]		RR=75% for the whole sample
Zhou [88]	56 / 173	unknown for subgroup*	female only	Self-developed survey items		Focus: sexual health & needs<br>RR=89% for the whole sample

	N	Potential sampling bias RR	% female	Potential methodological bias	Potential reporting bias	Other points of consideration
**Qualitative/ Mixed method studies**						
Bolte [72]	8	Follow-up from survey	38	1 phone focus group, guided by McGill Illness Narrative interview protocol, added 'sex life'		
Connell [64]	35	c		Home or phone interviews	Needs related to 'sexual issues' were not defined, but other identified needs may also include sex-related issues, but were also not defined	
Corney [70]	33 /105	unknown for subgroup*	female only	interviews	'Need for more information' was not further described/defined	RR=76% for the whole sample
Dobinson [73]	11	c	55	interviews		Focus: sexual health & needs
Gorman [74]	29	c	female only	Phone interviews		Focus: sexual health & needs<br>Also recruited *n*=25 male partners
Gould [75]	65	c	female only	10 focus groups		Focus groups stratified by relationship status and parent status
Lewis [76]	33	69%	female only	interviews		African American
Lopez [71]	19	not reported	female only	Phone interviews		Also recruited n=19 'old' survivors
Mattson [77]	337	52%	female only	Self-developed closed and open survey questions **		
Miedema [78]	53	c	74	Phone interviews		
Olsson [84]	23 / 44	unknown for subgroup*	not reported by subgroup#	6 in-person focus groups		RR=33% for the whole sample
Ruddy [79]	36	not reported	female only	4 focus groups		
Ruddy [80]	20	c	female only	Phone interviews		
Thompson [81]	8	16%	38	1 focus group	Identified categories were not further defined/described	
Tsangaris [82]	8 / 20	unknown for subgroup*	not reported by subgroup#	Phone interviews based on the Supportive Care Needs Framework		Findings were discussed in light of a literature review as part of this study<br>RR=67% for the whole sample
Zebrack [83]	37	79%	not reported	Survey-based Delphi Panel, incl. 3 iterative rounds		Also recruited *n*=40 healthcare providers

c = convenience sampling; RR = response rate
* not indicated as red flag, given that the larger study did not specifically intended to recruit the subgroup we focused on in this review (and RR for the full sample are indicated)
** indicated in lighter shade, because any qualitative study develops/adjusts their own phrasing of open-ended questions; but in a direct conversation participants are able to clarify or react to certain questions, which is less possible in written form
# indicated as red flag, given that studies reported subgroup results, but did not describe their subgroups in detail

Online Resource References
(aligned with reference numbers in the manuscript)

24. Zebrack, B., Information and service needs for young adult cancer survivors. *Supportive Care in Cancer*, 2009; **17**(4): p. 349-357.
34. Graugaard, C., C.D. Sperling, B. HolgeHazelton, K.A. Boisen, and G.S. Petersen, Sexual and romantic challenges among young Danes diagnosed with cancer: Results from a cross-sectional nationwide questionnaire study. *Psycho-oncology*, 2018; **27**(6): p. 1608-1614.
54. Boyes, A., A. Girgis, and C. Lecathelinais, Brief assessment of adult cancer patients' perceived needs: development and validation of the 34-item Supportive Care Needs Survey (SCNS-SF34). *Journal of evaluation in clinical practice*, 2009; **15**(4): p. 602-606.
55. Hodgkinson, K., P. Butow, G. Hunt, S. Pendlebury, K. Hobbs, S.K. Lo, and G. Wain, The development and evaluation of a measure to assess cancer survivors' unmet supportive care needs: the CaSUN (Cancer Survivors' Unmet Needs measure). *Psycho-Oncology: Journal of the Psychological, Social and Behavioral Dimensions of Cancer*, 2007; **16**(9): p. 796-804.
56. Kedde, H., H.B.M. Van De Wiel, W.C.M. Weijmar Schultz, and C. Wijsen, Sexual dysfunction in young women with breast cancer. *Supportive Care in Cancer*, 2013; **21**(1): p. 271-280.
57. JonkerPool, G., H.J. Hoekstra, G.W. Van Imhoff, D.J.A. Sonneveld, D.T. Sleijfer, M.F. Van Driel, H.S. Koops, and H.B.M. Van De Wiel, Male sexuality after cancer treatment - Needs for information and support: Testicular cancer compared to malignant lymphoma. *Patient education and counseling*, 2004; **52**(2): p. 143-150.
58. Bender, J.L., D. Wiljer, M.J. To, P.L. Bedard, P. Chung, M.A.S. Jewett, A. Matthew, M. Moore, P. Warde, and M. Gospodarowicz, Testicular cancer survivors' supportive care needs and use of online support: A cross-sectional survey. *Supportive Care in Cancer*, 2012; **20**(11): p. 2737-2746.
59. Geue, K., R. Schmidt, A. Sender, S. Sauter, and M. Friedrich, Sexuality and romantic relationships in young adult cancer survivors: Satisfaction and supportive care needs. *Psycho-oncology*, 2015; **24**(11): p. 1368-1376.
60. Hall, A.E., A.W. Boyes, J. Bowman, R.A. Walsh, E.L. James, and A. Girgis, Young adult cancer survivors' psychosocial well-being: A cross-sectional study assessing quality of life, unmet needs, and health behaviors. *Supportive Care in Cancer*, 2012; **20**(6): p. 1333-1341.
61. Sender, A., M. Friedrich, K. Leuteritz, E. Nowe, Y. Stobel-Richter, A. Mehnert, and K. Geue, Unmet supportive care needs in young adult cancer patients: associations and changes over time. Results from the AYA-Leipzig study. *Journal of Cancer Survivorship*, 2019; **13**(4): p. 611-619.
62. Smith, A., M. King, P. Butow, T. Luckett, P. Grimison, G.C. Toner, M. Stockler, E. Hovey, J. Stubbs, G. Hruby, H. Gurney, S. Turner, M. Alam, K. Cox, and I. Olver, The prevalence and correlates of supportive care needs in testicular cancer survivors: A cross-sectional study. *Psycho-oncology*, 2013; **22**(11): p. 2557-2564.
63. Zebrack, B.J., R. Block, B. Hayes-Lattin, L. Embry, C. Aguilar, K.A. Meeske, Y. Li, M. Butler, and S. Cole, Psychosocial service use and unmet need among recently diagnosed adolescent and young adult cancer patients. *Cancer*, 2013; **119**(1): p. 201-214.
64. Connell, S., C. Patterson, and B. Newman, Issues and concerns of young Australian women with breast cancer. *Support Care Cancer*, 2006; **14**(5): p. 419-26.
65. Dyson, G.J., K. Thompson, S. Palmer, D.M. Thomas, and P. Schofield, The relationship between unmet needs and distress amongst young people with cancer. *Supportive Care in Cancer*, 2012; **20**(1): p. 75-85.
66. Park, B.W. and S.Y. Hwang, Unmet needs of breast cancer patients relative to survival duration. *Yonsei medical journal*, 2012; **53**(1): p. 118-125.
67. Sender, A., M. Friedrich, R. Schmidt, and K. Geue, Cancer-specific distress, supportive care needs and satisfaction with psychosocial care in young adult cancer survivors. *European journal of oncology nursing : the official journal of European Oncology Nursing Society*, 2020; **44**: p. 101708.
68. Gupta, A.A., K. Edelstein, A. AlbertGreen, and N. D'Agostino, Assessing information and service needs of young adults with cancer at a single institution: The importance of information on cancer diagnosis, fertility preservation, diet, and exercise. *Supportive Care in Cancer*, 2013; **21**(9): p. 2477-2484.
69. Mutsch, J., M. Friedrich, K. Leuteritz, A. Sender, K. Geue, A. Hilbert, and Y. StobelRichter, Sexuality and cancer in adolescents and young adults - A comparison between reproductive cancer patients and patients with non-reproductive cancer. *BMC Cancer*, 2019; **19**(1): p. no pagination.
70. Corney, R.H., M.E. Crowther, H. Everett, A. Howells, and J.H. Shepherd, Psychosexual dysfunction in women with gynaecological cancer following radical pelvic surgery. *British journal of obstetrics and gynaecology*, 1993; **100**(1): p. 73-78.
71. Lopez, A.-L.J., P.N. Butow, S. Philp, K. Hobbs, E. Phillips, R. Robertson, and I. Juraskova, Age-related supportive care needs of women with gynaecological cancer: A qualitative exploration. *European Journal of Cancer Care*, 2019; **28**(4): p. e13070.
72. Bolte, S., The impact of cancer and its treatments on the sexual self of young adult cancer survivors and as compared to their healthy peers, in Dissertation Abstracts International. *ProQuest Information & Learning; US* 2010. p. 2219.
73. Dobinson, K.A., M.A. Hoyt, Z.E. Seidler, A.L. Beaumont, S.E. Hullmann, and C.R. Lawsin, A Grounded Theory Investigation into the Psychosexual Unmet Needs of Adolescent and Young Adult Cancer Survivors. *Journal of Adolescent and Young Adult Oncology*, 2016; **5**(2): p. 135-145.

74. Gorman, J.R., E. Smith, J.H. Drizin, K.S. Lyons, and S.M. Harvey, Navigating sexual health in cancer survivorship: a dyadic perspective. *Supportive Care in Cancer*, 2020(pagination): p. no pagination.
75. Gould, J., P. Grassau, J. Manthorne, R.E. Gray, and M.I. Fitch, 'Nothing fit me': nationwide consultations with young women with breast cancer. *Health Expectations*, 2006; **9**(2): p. 158-173.
76. Lewis, P.E., M. Sheng, M.M. Rhodes, K.E. Jackson, and L.R. Schover, Psychosocial concerns of young African American breast cancer survivors. *Journal of Psychosocial Oncology*, 2012; **30**(2): p. 168-184.
77. Mattsson, E., K. Einhorn, L. Ljungman, I. SundstromPoromaa, K. Stalberg, and A. Wikman, Women treated for gynaecological cancer during young adulthood - A mixed-methods study of perceived psychological distress and experiences of support from health care following end-of-treatment. *Gynecologic oncology*, 2018; **149**(3): p. 464-469.
78. Miedema, B., J. Easley, and L.M. Robinson, Do current cancer follow-up care practices meet the needs of young adult cancer survivors in Canada? A qualitative inquiry. *Current Oncology*, 2013; **20**(1): p. 14-22.
79. Ruddy, K.J., M.L. Greaney, K. Sprunck-Harrild, M.E. Meyer, K.M. Emmons, and A.H. Partridge, Young Women with Breast Cancer: A Focus Group Study of Unmet Needs. *Journal of Adolescent and Young Adult Oncology*, 2013; **2**(4): p. 153-160.
80. Ruddy, K.J., M.L. Greaney, K. Sprunck-Harrild, M.E. Meyer, K.M. Emmons, and A.H. Partridge, A qualitative exploration of supports and unmet needs of diverse young women with breast cancer. *Journal of Community and Supportive Oncology*, 2015; **13**(9): p. 323-329.
81. Thompson, K., S. Palmer, and G. Dyson, Adolescents & young adults: Issues in transition from active therapy into follow-up care. *European Journal of Oncology Nursing*, 2009; **13**(3): p. 207-212.
82. Tsangaris, E., J. Johnson, R. Taylor, L. Fern, D. BryantLukosius, R. Barr, G. Fraser, and A. Klassen, Identifying the supportive care needs of adolescent and young adult survivors of cancer: A qualitative analysis and systematic literature review. *Supportive Care in Cancer*, 2014; **22**(4): p. 947-959.
83. Zebrack, B., A. Bleyer, K. Albritton, S. Medearis, and J. Tang, Assessing the health care needs of adolescent and young adult cancer patients and survivors. *Cancer*, 2006; **107**(12): p. 2915-2923.
84. Olsson, M., M. Jarfelt, P. Pergert, and K. Enskär, Experiences of teenagers and young adults treated for cancer in Sweden. *European Journal of Oncology Nursing*, 2015; **19**(5): p. 575-581.
85. McCarthy, M.C., R. McNeil, S. Drew, L. Orme, and S.M. Sawyer, Information needs of adolescent and young adult cancer patients and their parent-carers. *Supportive Care in Cancer*, 2018; **26**(5): p. 1655-1664.
86. Albers, L.F., S.F. Haj Mohammad, O. Husson, H. Putter, R.C.M. Pelger, H.W. Elzevier, and E. MantenHorst, Exploring Communication About Intimacy and Sexuality: What Are the Preferences of Adolescents and Young Adults with Cancer and Their Health Care Professionals? *Journal of Adolescent and Young Adult Oncology*, 2020; **9**(2): p. 222-238.
87. Aggarwal, R., K. Hueniken, L. Eng, S. Kassirian, I. Geist, K. Balaratnam, M. Liang, C.B. Paulo, A. Geist, P. Rao, L. Mitchell, A. Magony, J.M. Jones, S.C. Grover, M.C. Brown, J. Bender, W. Xu, G. Liu, and A.A. Gupta, Health-related social media use and preferences of adolescent and young adult cancer patients for virtual programming. *Supportive Care in Cancer*, 2020(pagination): p. no pagination.
88. Zhou, L., L. Qing, B. Shen, Z. Jin, H. Liu, and Y. Chen, Patterns and predictors of healthcare-seeking for sexual problems among cervical cancer survivors: An exploratory study in China. *Biomedical Research (India)*, 2017; **28**(14): p. 6355-6360.

+

91. Keegan, T.H.M., D.Y. Lichtensztajn, I. Kato, E.E. Kent, X.-C. Wu, M.M. West, A.S. Hamilton, B. Zebrack, K.M. Bellizzi, A.W. Smith, and A.Y.A.H.S.C.G. and the, Unmet adolescent and young adult cancer survivors information and service needs: a population-based cancer registry study. *Journal of Cancer Survivorship*, 2012; **6**(3): p. 239-250.
